# County augmented transformer for COVID-19 state hospitalizations prediction

**DOI:** 10.1038/s41598-023-36378-9

**Published:** 2023-06-20

**Authors:** Siawpeng Er, Shihao Yang, Tuo Zhao

**Affiliations:** grid.213917.f 0000 0001 2097 4943 H. Milton Stewart School of Industrial and Systems Engineering, Georgia Institute of Technology, Atlanta, GA 30332 USA

**Keywords:** Infectious diseases, Mathematics and computing

## Abstract

The prolonged COVID-19 pandemic has tied up significant medical resources, and its management poses a challenge for the public health care decision making. Accurate predictions of the hospitalizations are crucial for the decision makers to make informed decision for the medical resource allocation. This paper proposes a method named County Augmented Transformer (CAT). To generate accurate predictions of four-week-ahead COVID-19 related hospitalizations for every states in the United States. Inspired by the modern deep learning techniques, our method is based on a self-attention model (known as the transformer model) that is actively used in Natural Language Processing. Our transformer based model can capture both short-term and long-term dependencies within the time series while enjoying computational efficiency. Our model is a data based approach that utilizes the publicly available information including the COVID-19 related number of confirmed cases, deaths, hospitalizations data, and the household median income data. Our numerical experiments demonstrate the strength and the usability of our model as a potential tool for assisting the medical resources allocation.

## Introduction

Since its first outbreak in January 2020, COVID-19 has continued to spread with the emergence of many variants (alpha, delta, omicron and etc^1,2^). The spread of the COVID-19 has resulted in deaths and detrimental effect towards the economy^3,4^ in the United States. Policymakers in every states, and decision makers in companies, educational institutes and many other parties have tailored their decisions and resource allocation at different stages of COVID-19. As the pandemic progresses, the allocation of the medical resources becomes an important consideration for the policymakers, often based on the accurate predictions of the number of hospitalizations for every state.

With the concerted efforts from many parties^5–9^, researchers can assess to these publicly available data that are important for them to design their models. Such data include COVID-19 related information such as the confirmed cases, deaths, hospitalizations information, mobility, as well as other general information such as the demographics and the household median income data in every state and county. In COVID-19 related predictions, different research groups design different models based on their expertise. Examples of the available models include compartmental models such as the different variants of Susceptible-Infectious-Recovered (SIR) models^10–14^ and statistical models that use sophisticated regression approaches^15,16,16^. Besides, there are also computational simulation^17,18^ and deep learning models^19–21^ for predicting COVID-19 dynamics. Moreover, the Centers for Disease Control and Prevention (CDC) has been leading a collaborative effort to produce an ensemble model from different research groups^9,22^ (See more detailed discussions of the related work in COVID-19 dynamics prediction in a later section).

In the medical resources allocation, one of the key metrics used is the total number of hospitalizations. With accurate forecasts of the number of hospitalizations, decision makers can be well prepared for the incoming patients. Such accurate predictions can help them to make informed decision based on the available resources, and identify critical areas that need additional resources from the less severe areas. The prediction of the number of hospitalizations can be modelled as a time series prediction problem. We first collect COVID-19 related data for the past 7 days as the input, and design a model to predict the number of hospitalizations for the next four weeks. As we continue to obtain new data for each additional day, our model will update predictions for the next four weeks starting from the new date. Our model uses a fully data-driven predictive approach. Specifically, we build a self-attention deep learning model, which takes the input data from multiple sources and predicts the state level number of COVID-19-related hospitalizations for the future four weeks in the United States. We carefully evaluate our proposed method for different periods and compare our best model with other benchmark models to show its strength and usability.

In a time series prediction problem, there exists several choices of deep learning models, for example recurrent neural networks (RNN) such as LSTM-RNN^23^, and the more recent transformer models. Our proposed approach is based on a current state-of-art self-attention model in Natural Language Processing, also known as the transformer model. With the attention mechanism, a transformer model is able to capture both the short-term and long-term dependencies within the time series while enjoying computational efficiency. The self-attention module allows a transformer model to capture the dependencies on previous time steps by assigning attention scores. A large score between two events implies a strong dependency, while a small score implies a weak one. With such a scoring mechanism, the transformer model is able to capture both short-term and long-term dependencies by adaptively selecting time steps that are at any temporal distance from the current time step. Because of the non-recurrent structure of the transformer, it is easy for a transformer model to stack multiple attention layers without the risk of gradient explosion and gradient vanishing. The gradient explosion and gradient vanishing phenomena are common among RNN-based models, rendering such models more difficult to be trained. Stacking of multiple attention layers allows a transformer model to better capturing higher order dependencies, that is harder to be achieved in a shallow RNN-based model. Since the computation for any two time steps is independent of each other in a transformer model, computational efficiency can be achieved as full parallelism is allowed when calculating dependencies across all time steps.

Our goal in this paper is to predict the weekly total number of hospitalizations at the state level for the next four weeks, given the current week data. Our model predicts both the point estimation and the probabilistic distribution, with predictions for a total of 23 quantiles (from 0.01 to 0.99 according to the CDC submission requirement). Our input data includes the number of confirmed cases, the number of deaths, the household median income data, and the hospitalizations data. We build a self-attention model, also known as the transformer model in Natural Language Processing, that is able to capture both the short and long term dependencies within the input time series data. In additional, our model includes a residual connection^24^ that connects the embedding from input layer to the linear decoder layers. Our models has two main ideas. Firstly, we augment the state level training data with the county level data. Such an addition of county level training data provides critical training signal for the model to learn from the input data itself. Secondly, we include a residual connection in our transformer model. We find that such a residual connection helps to improve the overall quality of the predictions. Moreover, the inclusion of a residual connection is crucial for the first two weeks predictions, where a model without such a connection may predict worse than a Naive model. When compared with other benchmark models, our model shows strong performance across different periods, showing its strength and usability for the prediction of the COVID-19 related number of hospitalizations.

### Related work

There are four main classes of predictive models in the number of hospitalizations prediction: compartmental models, simulation modeling, statistical models, and deep learning models. At the CDC website, the final CDC predictions are obtained by ensembling predictions from all the submitted models^9,22^.

- *Compartmental model* characterizes the disease spread dynamics using systems of ordinary differential equations. Several research groups use Susceptible-Infectious-Recovered (SIR) model in the number of hospitalizations predictions. In the SIR^13,25,26^ model, the population of the area is assigned to Susceptible (S), Infectious (I), or Recovered (R) mode. Another variant of SIR model is the SEIR model^10,12,27,28^ which introduces additionally Exposed (E) mode. In these compartmental models, the transitions from one mode to another mode (i.e., the disease spreading dynamics) are modeled as differential equations, often in the form of a transition matrix. Compartmental models are often selected for good interpretability of their results^29,30^, and they require serious domain expertise to design accurate differential equations to capture the underlying disease transmission dynamics.^31^. On the other hand, research group^32^ may use discrete-time difference instead of ordinary differential equations to model the transition matrix.
- *Simulation modeling* is another modeling approach that uses computer simulation to model different components in the studied environment and observes their interactions. Two typical simulation modeling techniques are cellular automata^33^, and agent-based simulation^17,18^ where agent-based simulation is the more common choice for a complex system. Most simulation modelings require research groups to assess to the intensive computational resources, and the researchers may need to conduct multiple simulations^33^.
- *Conventional statistical models*, include ARIMA, Gaussian process regression, and linear regression use regression methods to fit the data directly. Such models are more flexible than the compartmental models. A statistical model often requires dedicated effort in feature engineering and input selections^16^. One of the very first statistical models used in COVID-19 related predictions is the CLEP model^15^ that uses an ensemble model of an exponential predictor and a linear predictor. One of the recent models for the number of hospitalizations prediction, the model from Ref.^16^ uses autoregressive model on the Google Search Data to make predictions.
- *Deep Learning models* are deep neural networks that learn directly from their input data. These models are highly flexible and take advantage of their representation capability. Such models need a less sophisticated handcrafting preprocessing of the input data. The nature of the time series prediction problem requires the deep learning models to have the ability to capture the intrinsic information from a sequential data. Some common deep learning models include Long short-term memory (LSTM)^23^, Gated Recurrent Unit (GRU)^34^, and transformer^35,36^. While being a highly flexible model with a powerful representation capability, a deep learning model often requires larger training data. Concurrently with our work, there are other deep learning models including models from Ref.^19^ and Ref.^21^ that utilize attention mechanism from transformer architecture in their predictions of the number of hospitalizations.

### Our contribution

Our goal in this paper is to predict the weekly total number of hospitalizations at the state level for the next four weeks, given the current week data. Our model predicts both the point estimation and as well as the probabilistic distribution, with predictions for a total of 23 quantiles (from 0.01 to 0.99 according to the CDC submission format). Our input data include the number of confirmed cases, the number of deaths, median income data, and hospitalizations data. We build a self-attention model that is able to capture both the short and long term dependencies within the time series input data. The main contributions of our paper are as follows:

1. We propose a novel application of the transformer model, which is primarily used in Natural Language Processing, to the problem of predicting COVID-19 related hospitalizations. The self-attention mechanism of the transformer model enables efficient and accurate capturing of short-term and long-term dependencies in the input time series data.
2. We introduce the concept of county augmentation, wherein we augment our state-level training data with county-level data. This addition of county-level training data provides a critical training signal for the model, allowing it to learn from the input data more effectively and improve prediction accuracy.
3. We incorporate a residual connection in our transformer model, which we found to significantly enhance the overall quality of predictions. The residual connection is particularly crucial for the first two weeks predictions, where a model without such a connection may fail to predict better than a Naive model.
4. Our extensive experiments demonstrate that our model outperforms several benchmark models across different periods, highlighting the strength and usability of our proposed method for predicting COVID-19 related hospitalizations. This accurate prediction can greatly assist decision-makers in allocating medical resources more effectively, ultimately benefiting public health.

By addressing the limitations of existing models and proposing novel techniques to improve prediction accuracy, our paper presents a valuable contribution to the field of COVID-19 hospitalizations prediction.

## Results

To evaluate our proposed method, we compare point predictions among several baseline models with mean absolute error (MAE) as our comparison metrics. We compare the predictions from our CAT model with a few baseline models to better understand the CAT model. All model details are listed below.

- *CAT* - our proposed model that uses both state and county level data for training, and with a residual connection that connects the embedding after the input layer to the linear decoder layer.
- *WR* - a sub-model that uses the same settings as the CAT model, but Without the Residual (WR) connection that connects the embedding after the input layer to the linear decoder layer. The contrast will help us to understand the power of residual connection.
- *STATE* - a sub-model that uses only state level training data for training, thus the name “STATE”. The STATE model uses the same settings as the CAT model, but during the training phase, we only use the state level data to train the model. The contrast will help us to understand the power of county-level data.
- *Naive* - a model-free approach that does not require any training and it simply uses the current week’s reported total number of hospitalizations as the predictions for the next four weeks. The contrast will evaluate the predictive power in addition to the time series persistence.

In the CAT and WR models, county augmentation is performed by incorporating county-level data (and predictions) during the training phase. This approach enables the models to leverage the additional granularity and data size provided by county-level data in order to enhance the overall prediction performance. During the data preprocessing, we aggregate the county-level data and combine it with the state-level data to create an expanded dataset. Both the CAT and WR models are then trained on this combined dataset, effectively utilizing the county-level data augmentation in their training phases. The CAT model benefits from the county level augmentation as compared to the STATE model, and further benefits from the residual connection as compared to the WR model. We separate our predictions according to the training period, corresponding to $50\%$, $60\%$, $70\%$, and $80\%$ of the total dataset, where we present the performance of each model across the non-overlapping periods in Table 1. We follow by comparing our model with the models at the COVID-19 forecast hub website^9^. Besides point prediction comparison, we compare quantile predictions with the available models at the COVID-19 forecast hub website. We use the weighted interval score (WIS)^37,38^ for the quantile prediction comparison. There are two groups of the available models at the forecast hub, with their forecasting dates differ by 1 day. We present comparisons of our model with CDC baseline models using Table 2 for point prediction and Table 3 for the quantile prediction comparison. We present the full comparisons, including different constituent models inside the COVIDhub ensemble, using Tables S1, S2, S3 and S4 at the Supplementary section. Among the models, the Hub-Baseline is a naive method based model, and the COVIDHub-CDC-ensemble and COVIDhub-trained-ensemble are both weighted ensemble of different constituents. The models submitted to COVID Hub encompass all different types of methods in the Related Work, and we refer the readers to Table S5 and reference^9^ for the details of their implementations. In general, all methods proposed have different performances for different prediction time intervals. However, COVIDhub ensemble model tends to have best overall performance among all benchmark models, and is thus highlighted here as our main benchmark.

### Point prediction at different periods

**Figure 1:**
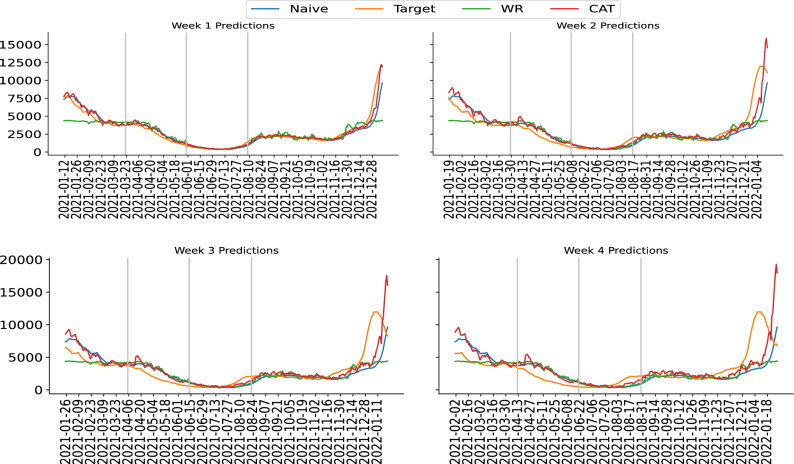
New York’s weekly total number of hospitalizations for Week 1 (upper left) predictions, Week 2 (upper right) predictions, Week 3 (lower left) predictions, and Week 4 (lower right) predictions. Vertical lines separate different prediction periods as in Table 1. “Target” is the true reported number of hospitalizations of New York. “CAT” is our proposed model. “WR” is the model without the residual connection. “Naive” is the Naive model prediction. More plots for other major states are presented in Supplementary Information.

We show the number of hospitalizations point prediction for different periods in Table 1. Since we use different amount of data points as our training set, we can take the non-overlapping period from each testing set as a separate out of the sample prediction period. CAT model can provide better predictions than Naive models in all periods. This model also performs the best in most prediction periods across different models. WR model is less consistent in its performance across different non overlapping periods, especially at the first two weeks predictions where it performs worse than the Naive model. We can assume WR model is focusing on the Week 3 and Week 4 predictions, than a more balanced and accurate prediction in CAT model. We also train our model without the county level data and present the model as STATE model. STATE model, similar to WR model, produce less consistent predictions across different prediction intervals. As more training data provided, CAT model continues to learn better and provides overall better predictions. We illustrate our predictions across different intervals using Fig. 1 and additional plot Fig. S2 in the Supplementary section. CAT model is able to produce better predictions than all other models, with the red line (CAT model) following more closely orange line (Target) in most of the periods.

**Table 1:** Different prediction periods for the weekly total number of hospitalizations.

Training intervals	Prediction intervals	Method	Week 1	Week 2	Week 3	Week 4
2020-05-02 to 2021-01-03	2021-01-04 to 2021-03-14	CAT	166.18	325.45	454.54	570.93
		WR	336.12	385.02	470.92	567.53
		STATE	524.65	491.93	488.68	502.58
		Naive	188.46	357.94	510.26	636.22
2020-05-02 to 2021-03-14	2021-03-15 to 2021-05-22	CAT	66.98	134.48	205.78	262.82
		WR	104.91	169.06	237.79	296.49
		STATE	81.56	127.44	181.69	229.46
		Naive	81.57	144.14	199.09	245.24
2020-05-02 to 2021-05-22	2021-05-23 to 2021-07-31	CAT	93.84	204.07	336.10	483.83
		WR	105.01	215.41	352.61	504.09
		STATE	121.79	240.50	386.50	543.69
		Naive	125.28	265.39	418.07	572.04
2020-05-02 to 2021-07-31	2021-08-01 to 2022-01-01	CAT	152.09	303.76	462.52	617.66
		WR	233.77	354.77	488.38	613.61
		STATE	256.15	380.07	513.14	636.30
		Naive	206.99	401.12	575.11	723.45

The prediction metrics reported is MAE.

### Point prediction comparison with benchmark models

We compare CAT model with the models at the COVID-19 forecast hub website. We first take the non overlapping forecast date among different portion of training dataset. From there, we compare the forecasting performance among different models with the same forecast dates. We found that there are two groups of models with their forecast dates differ by 1 day. We present the comparison of our model with the COVID-19 forecast hubs baseline models using Table 2 and present the full comparison using Tables S1 and S2 in the Supplementary section. We rank the models by the average of the prediction performance for four weeks. In all the predictions intervals, CAT model outperforms all the baseline models.

**Table 2:** Point prediction of hospitalizations for different models at different forecast intervals in terms of mean absolute error (MAE).

Prediction intervals	Method	Week 1	Week 2	Week 3	Week 4
2021-01-04 to 2021-03-08	CAT	161.1100	321.5719	462.8001	595.8786
	COVIDhub-CDC-ensemble^9^	202.3043	336.7702	485.7064	621.6681
	Hub-Baseline^9^	230.2340	349.3596	491.2659	619.9851
	Naive	190.7447	357.8362	519.3383	655.3255
2021-03-15 to 2021-05-17	CAT	68.3769	135.1611	205.0764	262.7986
	Naive	82.0596	145.1532	199.0277	246.1851
	COVIDhub-CDC-ensemble^9^	92.2255	145.9404	199.5511	241.6404
	Hub-Baseline^9^	125.7149	181.3617	232.0106	277.8106
2021-05-24 to 2021-07-26	CAT	88.2248	195.2382	325.8849	473.8845
	Naive	117.1936	251.3085	401.5298	555.8957
	COVIDhub-trained-ensemble^9^	121.4660	248.0660	399.9468	558.8745
	COVIDhub-CDC-ensemble^9^	133.3979	260.3340	412.8787	572.0617
	Hub-Baseline^9^	147.1809	274.6021	420.5809	571.3851
2021-08-02 to 2021-12-27	CAT	145.3961	290.3554	439.6925	591.5398
	Naive	201.8472	394.8433	572.2553	718.7079
	COVIDhub-CDC-ensemble^9^	214.1973	396.3191	575.9497	733.2573
	COVIDhub-trained-ensemble^9^	233.6248	399.0174	575.8936	733.2747
	Hub-Baseline^9^	258.9778	445.3337	613.9816	756.4381

### Quantile prediction comparison with benchmark models

In order to assess probabilistic forecast accuracy, the weighted interval score (WIS) is a proper score that combines a set of prediction interval score and can be interpreted as a generalization of the absolute error to probabilistic forecasts^37^. A smaller WIS indicates a better performance. WIS is defined as

$$\begin{aligned} \textrm{WIS}_{\alpha _{\{0: K\}}}(F, y)=\frac{1}{K+1 / 2} \times \left( w_{0} \times |y-m|+\sum _{k=1}^{K}\left\{ w_{k} \times {\text {IS}}_{\alpha _{k}}(F, y)\right\} \right) \end{aligned}$$

In the above equation, *K* is the number of prediction interval, with $\alpha _k$ is the coverage of the prediction interval, $w_{0}, w_{k}$ are the weights, IS is the interval score of a given observation *y* by the forecast *F*.

We compare our predictions with the available predictions at the COVID-19 forecast hub website in term of weighted interval score (WIS). Similar to the point prediction comparison, We present the comparison of our model with the COVID-19 forecast hubs baseline models using Table 3 and present the full comparison using Tables S3 and S4 in the Supplementary section. We rank the models by the average of the prediction performance for four weeks. In all the predictions intervals, CAT model outperforms all the baseline models.

**Table 3:** Prediction of hospitalizations for different models at different forecast intervals in terms of weighted interval score (WIS).

Prediction intervals	Method	Week 1	Week 2	Week 3	Week 4
2021-01-04 to 2021-03-08	CAT	101.2701	195.1030	291.2037	381.1156
	Hub-Baseline^9^	171.8772	228.0203	314.5714	407.2351
	COVIDhub-CDC-ensemble^9^	132.4239	220.0190	332.1019	445.7753
2021-03-15 to 2021-05-17	CAT	46.1973	85.8555	126.0659	158.2434
	COVIDhub-CDC-ensemble^9^	66.8859	95.0672	129.3859	160.1336
	Hub-Baseline^9^	124.7826	146.6162	170.7729	193.1620
2021-05-24 to 2021-07-26	CAT	71.1846	153.7234	260.0116	379.9787
	Hub-Baseline^9^	128.5375	205.0822	307.1347	420.4822
	COVIDhub-CDC-ensemble^9^	85.3851	191.8497	333.7217	487.2623
	COVIDhub-trained-ensemble^9^	83.4784	181.7284	420.4202	474.6177
2021-08-02 to 2021-12-27	CAT	109.1547	214.5480	326.6200	430.4874
	COVIDhub-CDC-ensemble^9^	149.5939	280.9419	428.9707	566.2292
	COVIDhub-trained-ensemble^9^	163.8719	293.9078	443.3500	582.9796
	Hub-Baseline^9^	185.7358	316.1545	448.9840	567.2913

## Discussion

From our result, we can see that CAT is a strong model that can produce accurate hospitalizations predictions for all 4 weeks across different periods. This shows that we could use this model across different periods of time. Our model is build upon two main ideas, that are augmented training signal from the county level data, and the inclusion of a residual connection in the model.

Our goal is to predict the state level hospitalizations, so it is natural to use the state level data as our training data. The STATE model is the model that uses only the state level data in its training. However, in the limitation of the available data, the STATE model will perform worse than that of Naive model. When we augment our training data with the county level data, the additional training signal helps to improve the model’s predictions. With the addition of the county level data, the WR model is able to produce better predictions than the STATE model. This justify our first idea of data augmentation using the county level data in our training process. Nevertheless, the predictions from the WR model are not always more accurate than that of Naive model for all the four weeks. We observe WR model is particularly struggling at the Week 1 predictions, and have better Week 3 and Week 4 predictions than that of Naive model. The reason may be due to the signal from the encoder is more useful for the Week 3 and Week 4 predictions. Because of that, we include a residual connection to bypass the encoder in the original model. Our final model, CAT model is able to produce accurate predictions across different prediction intervals for all the four weeks predictions. Moreover, for most of the predictions where other models perform better than that of Naive model, CAT model can further improve the overall prediction performance. In summary, the CAT model outperforms the STATE model by leveraging county-level augmentation, and it further surpasses the WR model by incorporating the residual connection, thus demonstrating the benefits of both proposed techniques in enhancing prediction performance. Other baseline models, such as COVIDhub-CDC-ensemble, COVIDhub-trained-ensemble, and Hub-Baseline, are models for which only prediction results are directly submitted to the CDC, limiting our ability to experiment with adding county augmentation or residual connection to these models.

While our CAT model shows strong results, one of the limitations of our current model is that the self-attention matrix from the encoder is not easily translated to an explainable pattern. The input of our model are the confirmed cases, deaths, household median income data and hospitalizations data. These inputs are encoded by the encoder, with self attention as a key mechanism to produce the final hidden representation for the decoders to produce the point predictions and the quantile predictions. In a transformer model, this attention representation is the weight matrix ($\dfrac{QK^T}{\sqrt{M_K}}$) in Equation (4). The practitioners of the transformer model^35^ may represent this weight matrix in a heatmap to visualize the relative importance of each factor on the final predictions. This is often the case if there exists a distinct pattern in such a heatmap. However, we do not find a distinct pattern that can be easily interpreted in this study. It will be beneficial to see how different factors contributes to the final predictions from the attention matrix.

As a deep learning model, CAT requires a large number of training dataset for the model to learn properly the trend. If there is a drastic change of trend, for example when there is an drastic increase then a drastic decrease, then the model may also fail to learn. In Fig. 1, we can see such a failure in the last period of time (December 14, 2021 to January 18, 2022). The trend is an increase that spans for several weeks before a huge decrease happen (Target line, orange color). However, CAT fails to react towards such a change and continues to predict an increment (CAT line, red color). This may due to the new trend from the Omicron, causing an introduction of new latent factor that failed to be captured in the previous training data. It is a future direction to quickly adapt the model for turning points in COVID-19 time series data.

In this project, we aim to capture the short term effects (of current week input) towards the future four weeks of predictions. As such, we use only 7 days of inputs when making the predictions. As a direction of future work, we can make use of a longer time series data as input. It is possible to discover cyclic or seasonality effect from a longer inputs and improve the current model. Besides, our CAT model predicts the 4 weeks ahead predictions together. In our current setup, our model predicts the total number of weekly hospitalizations for four weeks at once. Another direction for future research involves making predictions in an auto-regressive manner, which will require modifying the decoder component of the current model. Specifically, the model will first produce week 1 predictions, then use these outputs as input to the decoder to generate predictions for the second week. This process will be repeated until predictions for all four weeks are obtained. However, the exploration of this auto-regressive prediction approach is outside the scope of the current study and will be left for future research. Another future research direction involves incorporating more relevant data at the county level, such as mobility patterns or Google search data^39^, to improve the model’s performance. Specifically, for mobility data, while we have not included it in the current study due to our expectations regarding the return-to-normal mobility trends following the introduction of vaccinations^40,41^, we propose exploring the integration of mobility data into our CAT model in the future. To account for potential changes in mobility patterns due to vaccinations, we can design a strategy to assign different weights to mobility data based on the time span or the vaccination status of the population. Overall, incorporating more relevant data could help our model capture other information that may exist in the data and potentially improve prediction performance. Finally, our model makes predictions only from the temporal data. In our future work, we plan to extend our work to include spatial information such as interaction among states, counties or major cities. We expect that the inclusion of the geographical information would further improving our model’s predictions.

## Methods

In this section, we present our data sources and data processing procedure used in this paper. We also present the details of our transformer-based model and our training procedure.

### Data sources

Three comprehensive datasets are used in this study, including the confirmed cases, deaths, household median income data and the hospitalizations data from four sources. This paper focuses on the states in the mainland of the United States and do not consider Hawaii, Alaska, and other unincorporated territories. We use data from 47 states and their corresponding counties.

*Confirmed cases and deaths of Covid-19*    We obtain the confirmed cases and deaths data from the JHU CSSE Covid-19 dataset^6^. The dataset is a publicly available curated dataset from different sources. We use data from January 22, 2020, to January, 2022. We use both the confirmed cases and deaths from every targeted states and their corresponding counties.

*Hospitalizations data*    We obtain hospitalizations data from HealthData.gov^42,43^. These data are two separated time series datasets, that are the state level time series data and the facility level time series data. We also obtain another state level time series data from the COVID Tracking Project^3^.

*Household median income data*    We obtain Year 2019 US household median income for every states from the official website of United States Census Bureau^44^.

### Data preparation

We identify input features required for the training of our model, including the number of confirmed cases, the number of deaths, the number of hospitalizations and the household median income information from our datasets. We consolidate all input data into state-level and county-level datasets. We also include the smoothed (averaged over past seven days) confirmed cases, deaths and hospitalizations as our input features.

For the hospitalizations data, we use three datasets from two sources. Both datasets have records for the number of hospitalizations. Prior to March 2021, COVID Tracking Project^3^ has a more complete initial data. They have less missing data and have earlier records. Public health data were carefully gathered and processed to produce the data that was closest to the real incidents^3^. The COVID Tracking Project stopped after March 2021. Subsequently, the official hospitalizations data that we use is from the HealthData.gov^42,43^. These data are from the official reports from all the hospitals (or similar facilities) from states and counties. We perform a regression to impute missing data prior March 2021 for the HealthData.gov datasets. Then we use the official hospitalization data from HealthData.gov throughout our project.

The total data are separated into training and testing datasets for each corresponding county and state. To test our method for different amount of data and time intervals, we use different amounts (50%, 60% and 70%, and 80%) of the total data as our training dataset, and the remaining data as our testing dataset. As we use multiple features as inputs, we apply standardization to the inputs to accommodate differences in scale for each input.

### Transformer-based model

The prediction of COVID-19 hospitalizations of a given sequence of input is a time series modeling problem. For a typical time series prediction, a sequence of previous days’ number of hospitalizations is given, and the goal is to predict the number of hospitalizations for the future day. In our current article, our prediction problem is different from this typical time series prediction setting. Our 11-dimensional input consists of the current week’s number of deaths, number of confirmed cases, smoothed (averaged over 7 days) number of confirmed cases, smoothed number of deaths, household median income data, total (adult and pediatric) hospitalizations, pediatric hospitalizations, adult hospitalizations, smoothed (averaged over 7 days) total hospitalizations, smoothed pediatric hospitalizations and smoothed total hospitalizations. That is, a single-day data vector $k_j \in {\textbf{R}}^{11}$. Instead of predicting the daily number of hospitalizations, our model predicts the weekly total number of hospitalizations for the next four weeks (Week 1, Week 2, Week 3 and Week 4), using only the current week (Week 0) input data. We are given a sequence $\{k_j\}_{j=1}^7$ of 7 days data, where each single-day data $k_j \in {\textbf{R}}^{11}$, occurs at time *j*. One current week data (Week 0) can be viewed as any 7 days data, e.g. from Sunday to Saturday. Then Week 1 is the next Sunday to Saturday, Week 2 is the second Sunday to Saturday , Week 3 is the third Sunday to Saturday, and Week 4 is the fourth Sunday to Saturday. Table S6 in the Supplementary section shows examples of dates for Week 0, Week 1, Week 2, Week 3 and Week 4. Week 0 is the current week and data from this week are the inputs, Week 1-4 are the future 4 weeks prediction date ranges. As the prediction weeks are continuous, we do not show the full list of dates in the table to prevent cluttering. In this article, our one day input data is a data vector of dimension 11 of the current week input information, and the weekly input for our COVID-19 prediction problem can be viewed as a sequence (7 days) of 11-dimensional vectors.

One key ingredient of the transformer-based model is the self-attention module. Unlike a RNN based model, the attention mechanism does not have a recurrent structure. In this work, we use the original positional encoding method^35^ to our data vector to incorporate the temporal information into the inputs. Besides, other positional encoding methods such as relative positional method^45^ can be used to provide the temporal information for each of single-day data vector in our input sequence.

The input sequence of single-day data vectors is first transformed using a matrix ${\textbf{U}} \in {\textbf{R}}^{M \times 11}$, which will later be learned during the training phase with $M=8$. After the transformation, for any single-day data $k_j$ and its corresponding time stamp *j*, the temporal vector $z_j$ and the single-day data vector ${\textbf{U}}k_j$ both reside in ${\textbf{R}}^M$. For the positional encoding, we precompute using the trigonometric functions to define a temporal encoding for each time stamp, $z_j \in {\textbf{R}}^M$^35^.

1 $$\begin{aligned} \left[ z_{\text{ j }}\right] _{2l}&=\sin \left( j/ 10000^{2 l / {M}}\right) \end{aligned}$$

2 $$\begin{aligned} \left[ z_{\text{ j }}\right] _{2l+1}&=\cos \left( j/ 10000^{2 l / {M}}\right) \end{aligned}$$

where $j \in \{1, 2, 3 \cdots 7\}$ is the position, and $l \in \{1, 2 , 3 \cdots 11\}$ is the dimension.

Given a sequence of 7 days data $\{k_j\}^7_{j=1}$, we get

3 $$\begin{aligned} {\textbf{X}} = (\textbf{UE} + {\textbf{Z}})^T, \end{aligned}$$

where ${\textbf{E}} = [k_1,k_2,\dots ,k_7] \in {{\textbf{R}}^{11 \times 7}}$ is a sequence of single-day data vectors, ${\textbf{Z}} = [z_1, z_2,\dots , z_7] \in \mathbf {M \times 7}$ is the concatenation of the temporal vectors.

Then, the ${\textbf{X}}$ is passed through the self-attention module and the attention output ${\textbf{S}}$ is computed by

4 $$\begin{aligned} {\textbf{S}} = \text {Softmax }\left( \dfrac{\textbf{QK}^{T}}{\sqrt{M_K}}\right) {\textbf{V}},\text { where }{\textbf{Q}} = \textbf{XW}^Q, {\textbf{K}} = \textbf{XW}^K, {\textbf{V}} = \textbf{XW}^V. \end{aligned}$$

Here ${\textbf{Q}}$, ${\textbf{K}}$, ${\textbf{V}}$ are the query, key and value matrices obtained from different linear transformations of ${\textbf{X}}$ with ${\textbf{W}}^Q$, ${\textbf{W}}^K \in {\textbf{R}}^{M \times M_K}$, ${\textbf{W}}^V \in {\textbf{R}}^{M \times M_V}$. ${\textbf{W}}^Q$, ${\textbf{W}}^K$ and ${\textbf{W}}^V$ are the respective weights for each linear transformation. Multi-head attention is often used in practice to increases the model flexibility and for a better data fitting. In the multi-head attention, different sets of weights $\{{\textbf{W}}^Q_h,{\textbf{W}}^K_h,{\textbf{W}}^V_h\}^H_{h=1}$ are used to compute different attention outputs ${\textbf{S}}_1,{\textbf{S}}_2,\dots ,{\textbf{S}}_H$. By concatenating all the attention outputs and passing through the final linear transformation, we obtain the final attention output

5 $$\begin{aligned} {\textbf{S}} = \left[ \mathbf {S_1},\mathbf {S_2},\dots ,\mathbf {S_H} \right] {\textbf{W}}^O \end{aligned}$$

where ${\textbf{W}}^O \in {\textbf{R}}^{HM_V \times M}$ is an aggregation matrix. In the experiment, $M_K$, $M_Q$, $M_V$ and *H* are all set to 8.

The self-attention mechanism allows the selection of any single-day data whose occurrence time is at any distance from the current time. The *j*-th column of the attention score from the Softmax$(\textbf{QK}^T/\sqrt{M_K})$ indicates the extent of dependency of *j*-th single-day data ($k_j$) on its history. As such, attention mechanism allows the capturing of short and long term dependencies of the sequence data. For RNN-based models, such models encode the data’s history sequentially via hidden representations of events. In RNN-based models’ representation, the state of *j* depends on that of $j-1$, which in turn depends on $j-2$, etc. At any point of time, when a RNN-based model fails to learn sufficient information for any single-day data at *j*, the subsequent hidden representation of any other single-day data at *t* where $t \ge j$ will be adversely impacted.

The attention output ${\textbf{S}}$ is passed through a position-wise feed forward neural network to generate a hidden representation ${\textbf{h}}(j)$ of the input data sequence:

6 $$\begin{aligned} {\textbf{H}} = \text {ReLU}(\mathbf {SW_1^{FF}} +{\textbf{b}}_1)\mathbf {W_2^{FF}} +{\textbf{b}}_2, \; {\textbf{h}}(j) = {\textbf{H}}(j,:). \end{aligned}$$

At the above equation, $\mathbf {W_1^{FF}} \in {\textbf{R}}^{M \times M_H}$, $\mathbf {W_2^{FF}} \in {\textbf{R}}^{M_H \times M}$, ${\textbf{b}}_1 \in {\textbf{R}}^{M_H}$,${\textbf{b}}_2 \in {\textbf{R}}^{M}$ are the corresponding weights and biases of the feed forward neural networks. The resulting matrix ${\textbf{H}} \in {\textbf{R}}^{7 \times M}$ encodes hidden representations of all the information in the input sequence, where each row corresponds to a particular information. This final representation is used as an input to the linear decoder layers and to obtain the predictions of the weekly total number of hospitalizations for next four weeks. We set $M_H$ to 16 in the experiment. In our design, we have two linear decoders. Each decoder is a two layers network, with one decoder predicts the point estimation of the next 4 weeks, and the other decoder predicts the corresponding quantile predictions.

In a typical time series prediction, a model will forecast the next day prediction for a given current week data. In such a typical time series prediction, the model needs to have a masking for the attention mechanism to prevent “peeking into the future” issue. Such a masking allows any *j*-th data to attend only to any *t*-th data where $t \le j$. As compared, our model in this article predicts the weekly total number of hospitalizations for the next four weeks (Week 1, Week 2, Week 3 and Week 4), given the current week (Week 0) input data. This setting frees us from such a masking requirement since the model is implicitly masked from accessing the future total number of hospitalizations from the current week data.

In order to capture high level dependencies, a transformer based model also allows us to stack multiple self-attention modules together, and inputs are passed through each of these modules sequentially. However, such a stacking in RNN-based model is susceptible to gradient explosion and gradient vanishing, rendering the stacked model more difficult to train. Figure S1 in the Supplementary section illustrates the architecture of our transformer-based model used in this project.

### Residual connection

Residual network^24^ is a well established model in computer vision. Residual network (and its variants) contributes to the state of the art performance in computer vision. In a residual network, one of the main feature is the residual skip connection.

7 $$\begin{aligned} d = {\textbf{F}}(c, \{W_i\}) + c \end{aligned}$$

where *c* is the input, *d* is the output from the residual network, ${\textbf{F}}(c, \{W_i\})$ is the residual mapping to be learned. Residual skip connection can help to prevent vanishing gradient and accuracy degradation for deep models. The additional of a residual skip connection adds no additional parameters or computational cost. Residual skip connection also allows the model to have the choice to use the original identity mapping (*c*) or the output with additional transformation using the $W_i$. In our model, we connect the embedding after the input layer to the linear decoder layer using a residual connection.

### Training objective

Our network comprises of a shared encoder and two decoders. We design our problem as a multi-task learning with point estimation and quantile estimation as two separate tasks. Multi-task learning can help our model to learn better shared representations. Following common practices from the deep learning community^46,47^, we have two decoders and two loss functions. One decoder is used for point estimation. We train the transformer model for point estimation by using the Huber loss function^48^ . Specifically, the training objective is defined as

8 $$\begin{aligned} \text {min}\;{\textbf{L}} \left( f(h({\textbf{X}})),{\textbf{Y}} \right)&= \dfrac{1}{n}\sum _{i=1}^{n} z_{i}, \nonumber \\ \text {where }z_{i}&= {\left\{ \begin{array}{ll} \dfrac{1}{2} (f(h(x_i)) - y_i)^2 , &{} \text {if } |f(h(x_i)) - y_i| \le \delta \\ \delta \; \left( |f(h(x_i)) - y_i| - \dfrac{1}{2} \delta \right) , &{} \text {otherwise} \end{array}\right. } \end{aligned}$$

For the decoder that performs quantile estimation, we use the quantile loss function^49^. The training objective is defined as

9 $$\begin{aligned} \text {min}\;{\textbf{Q}} \left( g(h({\textbf{X}})),{\textbf{Y}} \right)&= \dfrac{1}{n}\sum _{i=1}^{n} \dfrac{1}{23}\sum _{j=1}^{23} r_{ij},\nonumber \\ \text {where }r_{ij}&= {\left\{ \begin{array}{ll} \alpha _j \left( y_i - g(h(x_i))\right) , &{} \text {if } y_i -g(h(x_i)) \ge 0 \\ \left( \alpha _j - 1\right) \left( y_i - g(h(x_i))\right) , &{} \text {otherwise} \end{array}\right. } \end{aligned}$$

The final objective function for our network is

10 $$\begin{aligned} {\text {min}}\;\ell = {\textbf{L}} + \beta {\textbf{Q}} \end{aligned}$$

In the above equations, our model’s shared encoder is represented by *h*, and the corresponding linear decoders are represented by *f* and *g*. In both the quantile loss and Huber loss functions, ${\textbf{X}}$ and ${\textbf{Y}}$ are the input space and the target space, with a pair of testing sample as $(x_i, y_i)$. We have *n* samples, $\alpha _j$ is a quantile. Following the CDC’s report standard, we have 23 different quantiles $\alpha _j \in$ {0.010, 0.025, 0.050, 0.100, 0.150, 0.200, 0.250, 0.300, 0.350, 0.400, 0.450, 0.500, 0.550, 0.600, 0.650, 0.700, 0.750, 0.800, 0.850, 0.900, 0.95, 0.975, 0.990}. In the huber loss function^48^, $z_i$ is the loss obtained from the *i*-th sample input $x_i$ and sample output $y_i$ using the huber loss function. $x_i$ is the input and has the form of $x_i \in {\textbf{R}}^{11 \times 7}$. $y_i$ is the 4 weeks hospitalizations ground truth of the future, and has the form $y_i \in {\textbf{R}}^{4}$. In the quantile loss function^49^, $r_{ij}$ is the loss obtained from the *i*-th sample input $x_i$, sample output $y_i$, and *j*-th quantile $\alpha _j$. $x_i$ and $y_i$ has the same form as the huber loss’s definition. Both $\delta$ and $\beta$ are tuning hyperparameters. In our experiment, we set $\delta$ to 1.0 and $\beta$ to 3.0. From a statistical perspective, the $\beta {\textbf{Q}}$ part of the network can be viewed as a regularizer, which helps to ensure the encoder to capture useful information both for the point prediction, as well as for the distribution estimation.

### Training details

**Figure 2:**
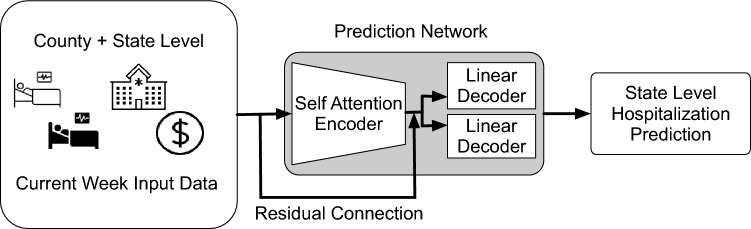
Overview of the Prediction Flow. The state-level predictions for weekly hospitalizations are for the next 4 weeks from the current week (Week 0) input data.

The transformer used in this paper has an encoder model dimensions of 8, 1 encoder layer with 8 attention heads and 16 feed forward dimensions. We connect the encoder layer’s output to a linear layer decoder for predicting weekly hospitalizations for the next 4 weeks using the current week input data for the point predictions. We connect the output from the encoder layer to another linear layer decoder for the quantile predictions. We have a residual skip connection from the input layer to each of the decoder layers. We use Adam^50^ optimizer and set 0.0075 as our initial learning rate, with a batch size of 512 and decay the learning rate by half after running for 250 epochs. We run our model for a total of 500 epochs. During the training phase, the transformer-based model will predict both county-level and state-level hospitalizations, with the corresponding county level and state level training input. Upon the completion of the training, we use our model to predict the state level hospitalizations for the next 4 weeks. Following the convention at the CDC forecast website, our model outputs both point predictions and predictions at different quantiles (total number of quantiles is 23, ranging from 0.01 to 0.99). Figure 2 illustrates our training process.

### Ethics approval and consent to participate

This study did not involve human participants, data, or tissue. It was conducted using only aggregated and anonymized data. Institutional review board approval was not required.

## Conclusion

In summary, this article presents the new model CAT for COVID-19 hospitalizations predictions at the state level for the United States. We use county-level data to provide additional training signal to our model. We include a residual connections in our transformer model to produce accurate prediction of COVID-19’s related hospitalizations. While we are in the process of recovering from COVID-19, resource allocation due to COVID-19 is still a challenging task. We hope through our model, we can improve the hospitalizations prediction and continue to provide insight for resource allocation and disease control planning.

## Supplementary Information

Supplementary Information.

## Data Availability

The datasets generated and/or analysed during the current study are publicly available in a GitHub Repository at https://github.com/esppeace/Covid_Hospitalizations.
