# Supplementary material for: County augmented transformer for COVID-19 state hospitalizations prediction
Source: Sci Rep. 2023 Jun 20;13:9955. doi: 10.1038/s41598-023-36378-9 (PMC10282074; doi:10.1038/s41598-023-36378-9)
Supplement: Supplementary file 1 — Supplementary Information. [file 41598_2023_36378_MOESM1_ESM.pdf]

Supplementary Information

Table S1. Point prediction of hospitalizations for different models at different forecast intervals for Group 1 models in term of mean absolute error (MAE).

Table S2. Point prediction of hospitalizations for different models at different forecast intervals for Group 2 models in terms of mean absolute error (MAE).

Table S3. Prediction of hospitalizations for different models at different forecast intervals for Group 1 models in terms of weighted interval score (WIS).

Table S4. Prediction of hospitalizations for different models at different forecast intervals for Group 2 models in terms of weighted interval score (WIS).

Table S5. Model types for different models in the Covid Hub[1].

Table S6. Examples of predictions date ranges for Week 0, Week 1, Week 2 and Week 4. Week 0 is the current week and data from this week act as input, Week 1-4 are the future 4 weeks prediction date ranges.

Fig S1. Transformer based model architecture.

Fig S2. Total number of hospitalizations for Week 1 (upper left) predictions, Week 2 (upper right) predictions, Week 3 (lower left) predictions, and Week 4 (lower right) predictions for Illinois, California, Texas, and Arizona. Vertical lines separate different prediction periods as in Table 1 in main article."Target" is the true reported number of hospitalizations of each state. "CAT" is our proposed model. "WR" is the model without the residual connection. "Naive" is the Naive model prediction.

Supporting information

Prediction Intervals	Method	Week 1	Week 2	Week 3	Week 4
2021-01-04 to 2021-03-08	LANL-GrowthRate[2]	278.0376	261.3700	335.8035	451.3300
	CAT	161.1100	321.5719	462.8001	595.8786
	COVIDhub-CDC-ensemble[1]	202.3043	336.7702	485.7064	621.6681
	JHUAPL-SLPHospEns[3]	210.8522	356.5968	503.9053	640.7427
	Hub-Baseline[1]	230.2340	349.3596	491.2659	619.9851
	Naive	190.7447	357.8362	519.3383	655.3255
	GT-DeepCOVID[4]	299.7154	436.7512	563.9064	678.5568
	JHUAPL-Bucky[5]	459.1361	528.5707	621.1138	714.1978
	MOBS-GLEAM-COVID[6]	292.9528	406.3794	538.8884	-
2021-03-15 to 2021-05-17	CAT	68.3769	135.1611	205.0764	262.7986
	Naive	82.0596	145.1532	199.0277	246.1851
	GT-DeepCOVID[4]	109.8397	149.1006	188.7413	229.7395
	COVIDhub-CDC-ensemble[1]	92.2255	145.9404	199.5511	241.6404
	LANL-GrowthRate[2]	173.5389	161.1929	170.5749	196.0712
	JHUAPL-SLPHospEns[3]	103.0443	157.5687	209.7350	251.8132
	Hub-Baseline[1]	125.7149	181.3617	232.0106	277.8106
	JHUAPL-Bucky[5]	189.3766	238.8872	285.4622	320.8425
2021-05-24 to 2021-07-26	CAT	88.2248	195.2382	325.8849	473.8845
	Naive	117.1936	251.3085	401.5298	555.8957
	COVIDhub-trained-ensemble[1]	121.4660	248.0660	399.9468	558.8745
	COVIDhub-CDC-ensemble[1]	133.3979	260.3340	412.8787	572.0617
	JHUAPL-SLPHospEns[3]	139.1027	267.9981	421.0844	580.2860
	Hub-Baseline[1]	147.1809	274.6021	420.5809	571.3851
	GT-DeepCOVID[4]	148.9143	274.3558	421.4868	572.4015
	MOBS-GLEAM-COVID[6]	148.5342	271.9365	421.7581	579.7784
	JHUAPL-Bucky[5]	152.7652	281.0585	430.8269	584.9627
2021-08-02 to 2021-12-27	CAT	145.3961	290.3554	439.6925	591.5398
	Naive	201.8472	394.8433	572.2553	718.7079
	COVIDhub-CDC-ensemble[1]	214.1973	396.3191	575.9497	733.2573
	COVIDhub-trained-ensemble[1]	233.6248	399.0174	575.8936	733.2747
	GT-DeepCOVID[4]	240.6978	412.9640	584.5282	728.7692
	Hub-Baseline[1]	258.9778	445.3337	613.9816	756.4381
	JHUAPL-Bucky[5]	376.6557	515.1448	664.5220	807.4207

Table S1. Point prediction of hospitalizations for different models at different forecast intervals for Group 1 models in terms mean absolute error (MAE).

Prediction Intervals	Method	Week 1	Week 2	Week 3	Week 4
2021-01-10 to 2021-03-14	CAT	110.7538	256.5210	375.8103	482.4043
	JHU-IDD-CovidSP[7]	337.6811	305.5340	338.2851	408.3509
	Karlen-pypm[8]	193.1889	296.0570	427.8446	550.9672
	Naive	186.3638	355.3511	496.4447	612.2511
	USC-SI-kJalpha[9]	221.5506	373.1894	510.5889	629.0438
	CU-scenario-mid[10]	252.6043	420.9702	559.6809	673.3000
	CU-select[10]	252.7638	421.1638	559.8702	673.4851
	CU-scenario-low[10]	252.7936	421.2574	559.9000	673.5787
	CU-scenario-high[10]	253.6574	421.9809	560.6830	674.3362
	CU-nochange[10]	253.5745	422.0426	560.7191	674.3468
	Covid19Sim-Simulator[11]	860.2411	1012.5447	1143.0741	1250.3451
2021-03-21 to 2021-05-16	CAT	60.7810	119.6276	188.4552	239.0694
	Karlen-pypm[8]	91.7894	124.2338	181.2655	238.4839
	Naive	81.9433	143.5910	198.8132	247.0591
	CU-scenario-low[10]	121.4090	168.4468	212.8463	246.5248
	CU-scenario-mid[10]	121.4634	168.5106	213.0567	246.7494
	CU-select[10]	121.4280	168.5887	213.0969	246.8605
	CU-nochange[10]	121.6123	168.6690	213.2057	246.6619
	JHU-IDD-CovidSP[7]	313.4676	299.7387	289.7503	283.0532
	Covid19Sim-Simulator[11]	308.5971	308.9057	316.2815	329.0476
2021-05-23 to 2021-07-25	Karlen-pypm[8]	87.2287	163.2232	304.6496	463.8602
	CAT	90.3571	202.8318	336.2214	484.4733
	JHUAPL-Gecko[12]	97.1833	216.2148	362.2728	517.9299
	USC-SI-kJalpha[9]	108.3254	212.6666	359.7763	516.6075
	Naive	113.0255	244.1894	392.0447	546.7574
	JHU-IDD-CovidSP[7]	226.8557	294.3749	399.5550	524.6044
	CU-select[10]	175.9872	299.2106	448.7936	606.0596
	CU-scenario-mid[10]	175.9936	299.1957	448.8000	606.0660
	CU-scenario-high[10]	176.0660	299.2596	448.8681	606.1213
	CU-scenario-low[10]	176.0447	299.4000	448.9617	606.2362
	CU-nochange[10]	176.2128	299.6447	449.0362	606.2766
2021-08-01 to 2021-12-05	CAT	121.4729	208.1953	295.5334	421.0862
	Naive	162.1680	289.6025	408.3427	548.3774
	JHUAPL-Gecko[12]	187.4184	279.3021	402.5020	556.4787
	USC-SI-kJalpha[9]	198.0788	291.0056	414.8159	571.4873
	Karlen-pypm[8]	237.0716	314.6812	432.0055	587.5764

Table S2. Point prediction of hospitalizations for different models at different forecast intervals for Group 2 models in terms of mean absolute error (MAE).

Prediction Intervals	Method	Week 1	Week 2	Week 3	Week 4
2021-01-04 to 2021-03-08	LANL-GrowthRate[2]	183.9253	183.0691	225.2389	295.8629
	CAT	101.5224	230.0362	354.8007	461.2456
	JHUAPL-SLPHospEns[3]	157.7405	222.6984	307.5640	397.8206
	Hub-Baseline[1]	171.8772	228.0203	314.5714	407.2351
	COVIDhub-CDC-ensemble[1]	132.4239	220.0190	332.1019	445.7753
	GT-DeepCOVID[4]	201.4844	314.1033	430.5716	541.6230
	JHUAPL-Bucky[5]	327.8737	388.3298	466.6548	549.9082
	MOBS-GLEAM-COVID[6]	201.8111	269.3117	356.9138	-
2021-03-15 to 2021-05-17	CAT	46.1973	85.8555	126.0659	158.2434
	LANL-GrowthRate[2]	102.7715	103.4680	113.2608	129.0114
	COVIDhub-CDC-ensemble[1]	66.8859	95.0672	129.3859	160.1336
	GT-DeepCOVID[4]	70.0935	99.3680	131.5625	163.1974
	JHUAPL-SLPHospEns[3]	75.7339	101.5759	131.2838	157.7032
	Hub-Baseline[1]	124.7826	146.6162	170.7729	193.1620
	JHUAPL-Bucky[5]	130.8531	155.8825	185.1444	210.4484
2021-05-24 to 2021-07-26	CAT	71.1846	153.7234	260.0116	379.9787
	MOBS-GLEAM-COVID[6]	87.8618	163.2165	281.9850	423.8713
	JHUAPL-SLPHospEns[3]	87.5107	179.5872	307.9520	453.1132
	JHUAPL-Bucky[5]	95.0125	183.2392	309.3552	451.5943
	Hub-Baseline[1]	128.5375	205.0822	307.1347	420.4822
	COVIDhub-CDC-ensemble[1]	85.3851	191.8497	333.7217	487.2623
	COVIDhub-trained-ensemble[1]	83.4784	181.7284	420.4202	474.6177
	GT-DeepCOVID[4]	104.3297	217.1641	356.3801	502.1687
2021-08-02 to 2021-12-27	CAT	109.1547	214.5480	326.6200	430.4874
	COVIDhub-CDC-ensemble[1]	149.5939	280.9419	428.9707	566.2292
	COVIDhub-trained-ensemble[1]	163.8719	293.9078	443.3500	582.9796
	Hub-Baseline[1]	185.7358	316.1545	448.9840	567.2913
	GT-DeepCOVID[4]	177.6375	326.3663	483.4453	619.7240
	JHUAPL-Bucky[5]	259.7981	345.7348	450.1775	557.0095

Table S3. Prediction of hospitalizations for different models at different forecast intervals for Group 1 models in terms of weighted interval score (WIS).

Prediction Intervals	Method	Week 1	Week 2	Week 3	Week 4
2021-01-10 to 2021-03-14	CAT	70.8006	157.3908	239.1504	314.6601
	Karlen-pypm[8]	153.6115	187.3175	249.0383	320.3509
	JHU-IDD-CovidSP[7]	243.5599	209.9301	219.8123	257.3966
	USC-SI-kJalpha[9]	186.0894	327.7555	463.4647	580.4688
	CU-scenario-low[10]	198.8377	358.3646	495.5887	608.9777
	CU-select[10]	199.2151	358.7731	495.9883	609.3282
	CU-scenario-mid[10]	199.2108	358.7926	496.0083	609.3461
	CU-nochange[10]	199.9963	359.7364	497.0335	610.4407
	CU-scenario-high[10]	200.0183	359.7143	496.9826	610.3140
	Covid19Sim-Simulator[11]	816.4972	967.4046	1096.9088	1204.1810
2021-03-21 to 2021-05-16	CAT	42.1237	77.9693	116.7653	146.4134
	Karlen-pypm[8]	80.5359	90.5123	112.8614	140.0275
	CU-select[10]	88.0350	128.8045	170.0385	202.8314
	CU-scenario-low[10]	88.1398	128.8598	170.0280	202.7401
	CU-scenario-mid[10]	88.0966	128.8458	170.0725	202.8214
	CU-nochange[10]	88.4811	129.1580	170.3218	202.9535
	Covid19Sim-Simulator[11]	230.2297	231.8630	233.4517	238.5503
	JHU-IDD-CovidSP[7]	256.3251	246.4041	235.5222	219.9566
2021-05-23 to 2021-07-25	Karlen-pypm[8]	65.1107	107.4737	210.2273	350.4126
	CAT	73.7608	159.8082	267.6992	388.7118
	JHUAPL-Gecko[12]	76.2683	160.6158	280.3231	415.5723
	USC-SI-kJalpha[9]	92.4183	194.0796	339.6717	496.2828
	JHU-IDD-CovidSP[7]	206.0139	255.2639	340.5163	449.0508
	CU-scenario-mid[10]	149.1729	269.0291	416.1379	572.8429
	CU-scenario-high[10]	149.2842	269.1239	416.1758	572.8697
	CU-select[10]	149.3938	269.3299	416.4070	573.1024
	CU-scenario-low[10]	149.4093	269.4085	416.4575	573.1755
	CU-nochange[10]	149.4671	269.4182	416.4367	573.1321
2021-08-01 to 2021-12-05	CAT	84.9991	136.9970	196.1271	287.2693
	Karlen-pypm[8]	163.0097	198.7800	262.1420	371.4978
	JHUAPL-Gecko[12]	131.2045	198.0741	297.6761	435.5240
	USC-SI-kJalpha[9]	143.6355	230.5367	347.5697	500.8673

Table S4. Prediction of hospitalizations for different models at different forecast intervals for Group 2 models in terms of weighted interval score (WIS).

Method	Type
LANL-GrowthRate[2]	Compartmental model
JHUAPL-SLPHospEns[3]	Simulation
Hub-Baseline[1]	Naive
COVIDhub-CDC-ensemble[1]	Weighted Ensemble
COVIDhub-trained-ensemble[1]	Weighted Ensemble
GT-DeepCOVID[4]	Deep learning model
JHUAPL-SLPHospEns[3]	Simulation
JHUAPL-Bucky[5]	Compartmental model
MOBS-GLEAM-COVID[6]	Simulation
Karlen-pypm[8]	Simulation
JHUAPL-Gecko[12]	Conventional statistical model
USC-SI-kJalpha[9]	Compartmental model
JHU-IDD-CovidSP[7]	Compartmental model
CU-nochange[10]	Compartmental model + Simulation
CU-select[10]	Compartmental model + Simulation
CU-scenario-low[10]	Compartmental model + Simulation
CU-scenario-mid[10]	Compartmental model + Simulation
CU-scenario-high[10]	Compartmental model + Simulation
Covid19Sim-Simulator[11]	Compartmental model

Table S5. Model types for different models in the Covid Hub[1].

Week 0	Prediction Generation Date	Week 1	Week 2	Week 3	Week 4
2020-12-27 to 2021-01-02	2021-01-02	2021-01-03 to 2021-01-09	2021-01-10 to 2021-01-16	2021-01-17 to 2021-01-23	2021-01-24 to 2021-01-30
2021-01-03 to 2021-01-09	2021-01-09	2021-01-10 to 2021-01-16	2021-01-17 to 2021-01-23	2021-01-24 to 2021-01-30	2021-01-31 to 2021-02-06
2021-01-10 to 2021-01-16	2021-01-16	2021-01-17 to 2021-01-23	2021-01-24 to 2021-01-30	2021-01-31 to 2021-02-06	2021-02-07 to 2021-02-13
2021-01-17 to 2021-01-23	2021-01-23	2021-01-24 to 2021-01-30	2021-01-31 to 2021-02-06	2021-02-07 to 2021-02-13	2021-02-14 to 2021-02-20
2021-01-24 to 2021-01-30	2021-01-30	2021-01-31 to 2021-02-06	2021-02-07 to 2021-02-13	2021-02-14 to 2021-02-20	2021-02-21 to 2021-03-27
2021-01-31 to 2021-02-06	2021-02-06	2021-02-07 to 2021-02-13	2021-02-14 to 2021-02-20	2021-02-21 to 2021-03-27	2021-02-28 to 2021-03-06

Table S6. Examples of predictions date ranges for Week 0, Week 1, Week 2 and Week 4. Week 0 is the current week and data from this week act as input, Week 1-4 are the future 4 weeks prediction date ranges.

Supplementary Figures

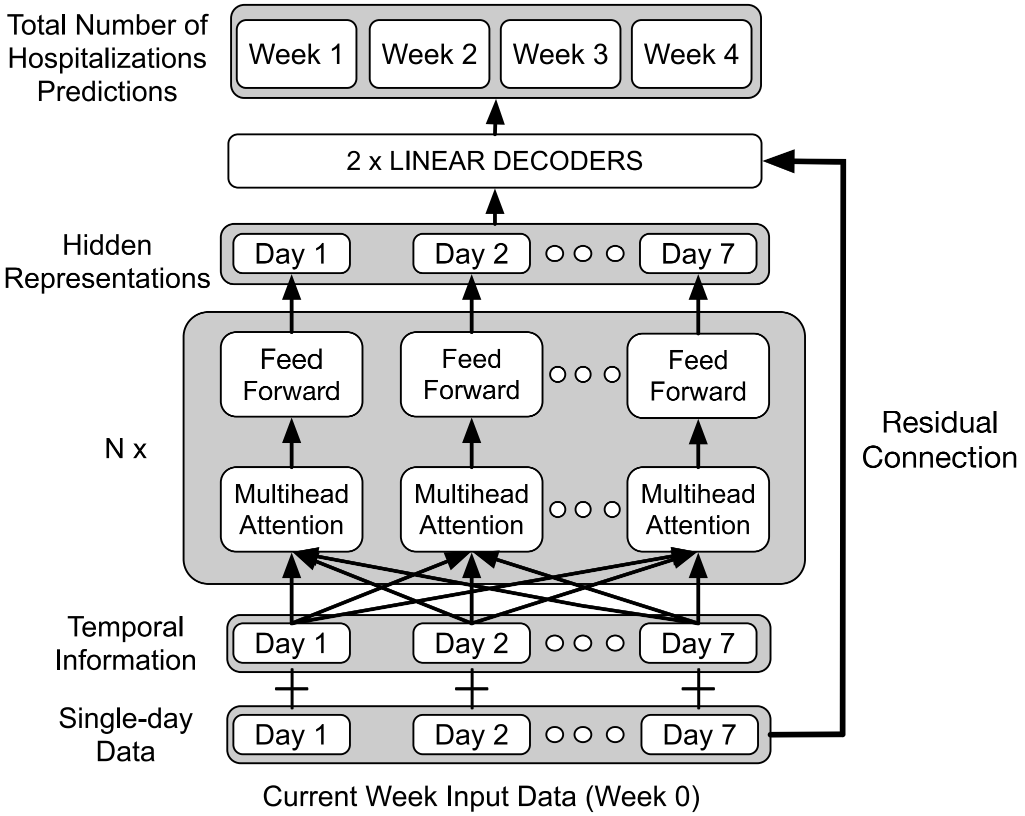

Figure S1. **Transformer based model architecture.**

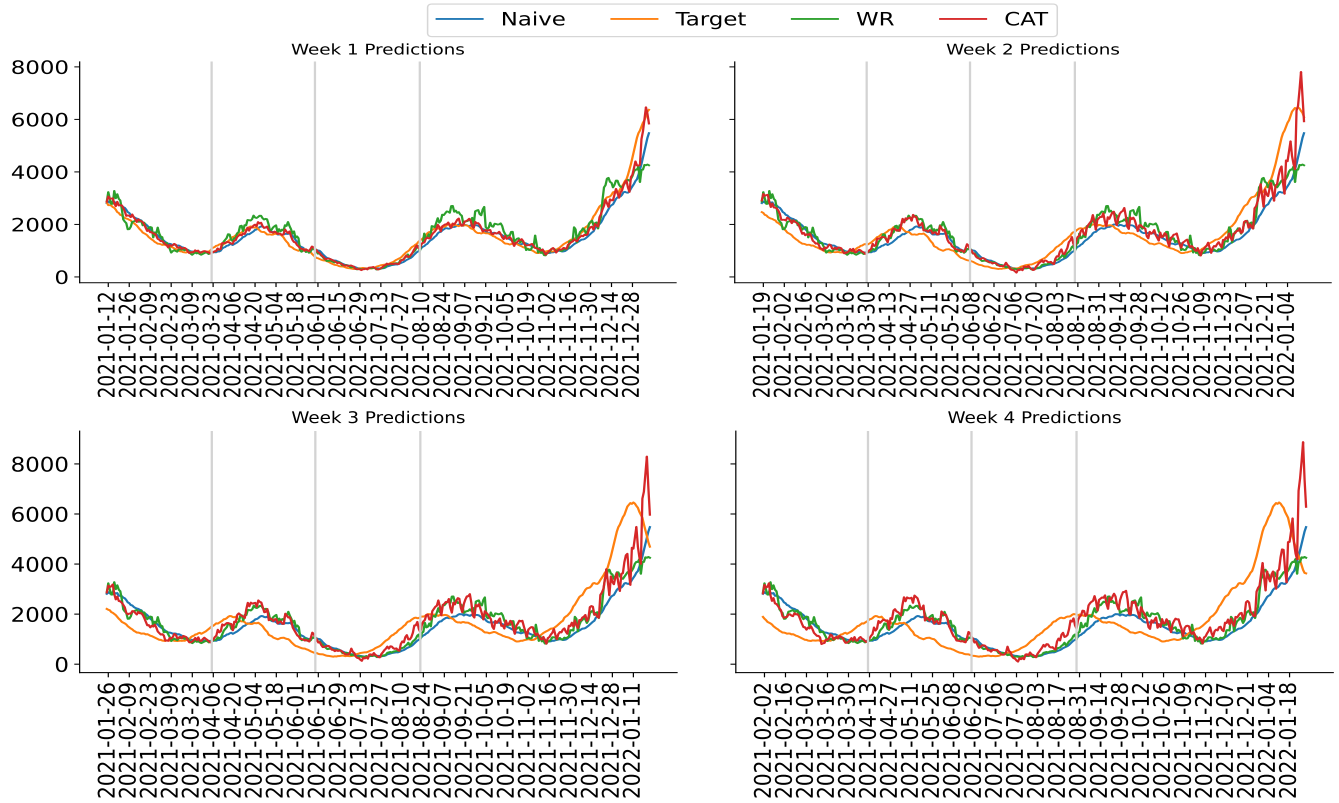

(a) Illinois

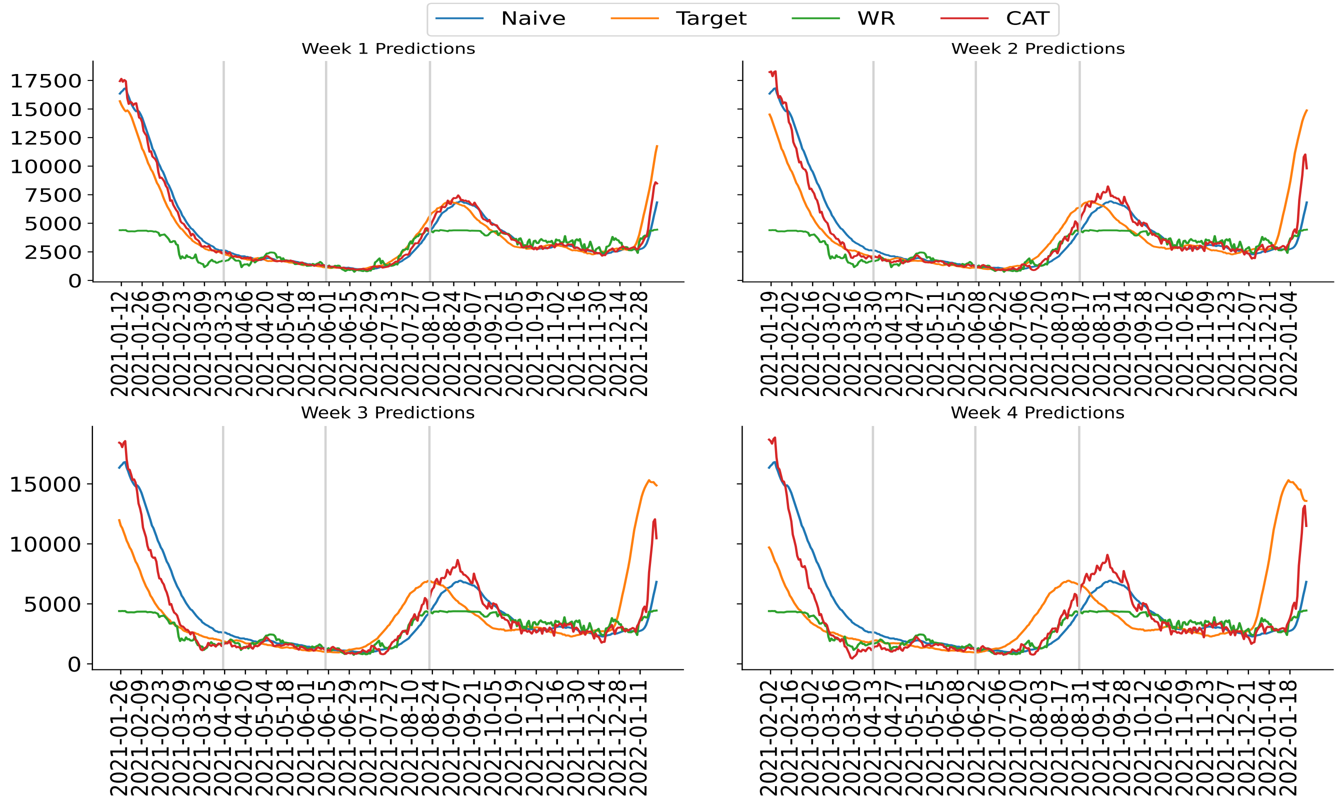

(b) California

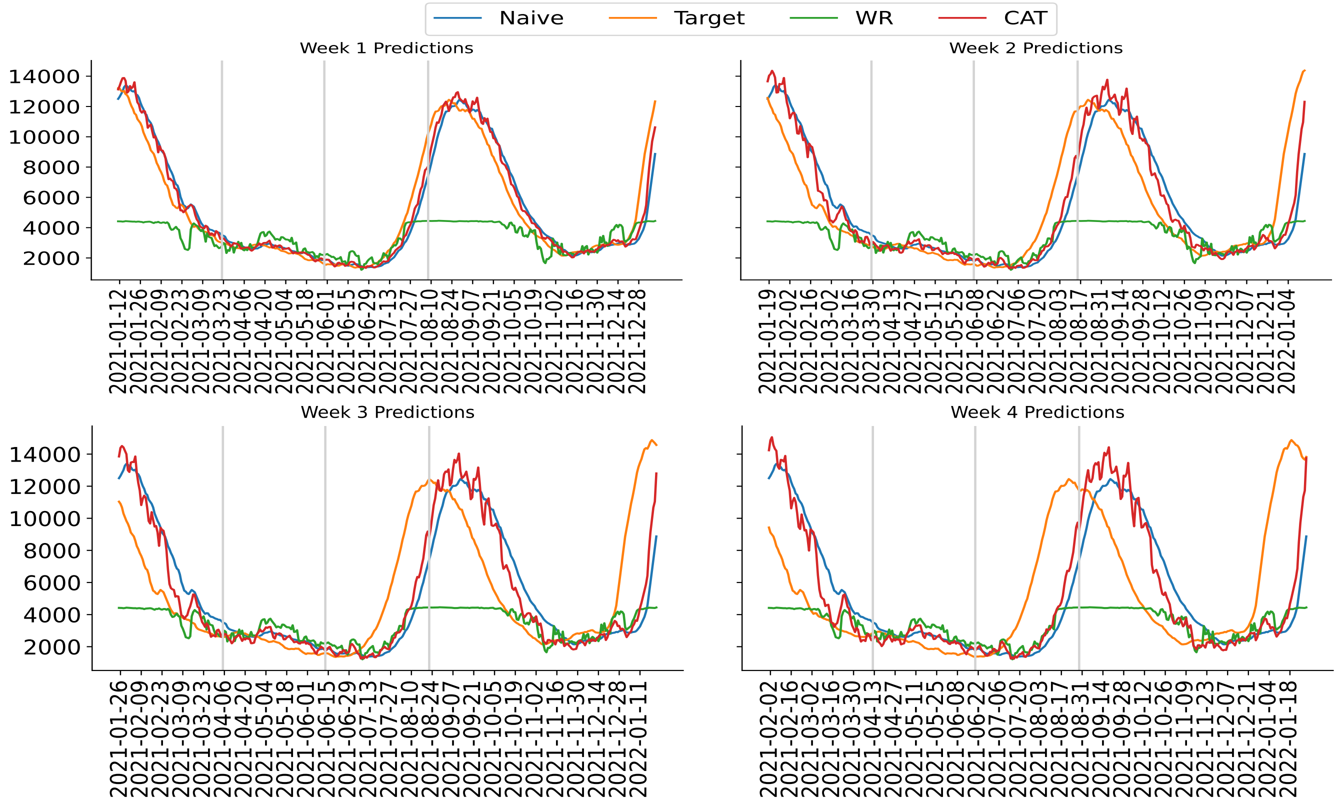
Naive
Target
WR
CAT
Week 1 Predictions
Week 2 Predictions
Week 3 Predictions
Week 4 Predictions
2000
4000
6000
8000
10000
12000
14000
2021-01-12 2021-01-26 2021-02-09 2021-02-23 2021-03-09 2021-03-23 2021-04-06 2021-04-20 2021-05-04 2021-05-18 2021-06-01 2021-06-15 2021-06-29 2021-07-13 2021-07-27 2021-08-10 2021-08-24 2021-09-07 2021-09-21 2021-10-05 2021-10-19 2021-11-02 2021-11-16 2021-11-30 2021-12-14 2021-12-28
2021-01-19 2021-02-02 2021-02-16 2021-03-02 2021-03-16 2021-03-30 2021-04-13 2021-04-27 2021-05-11 2021-05-25 2021-06-08 2021-06-22 2021-07-06 2021-07-20 2021-08-03 2021-08-17 2021-08-31 2021-09-14 2021-09-28 2021-10-12 2021-10-26 2021-11-09 2021-11-23 2021-12-07 2021-12-21 2022-01-04
2021-01-26 2021-02-09 2021-02-23 2021-03-09 2021-03-23 2021-04-06 2021-04-20 2021-05-04 2021-05-18 2021-06-01 2021-06-15 2021-06-29 2021-07-13 2021-07-27 2021-08-10 2021-08-24 2021-09-07 2021-09-21 2021-10-05 2021-10-19 2021-11-02 2021-11-16 2021-11-30 2021-12-14 2021-12-28 2022-01-11
2021-02-02 2021-02-16 2021-03-02 2021-03-16 2021-03-30 2021-04-13 2021-04-27 2021-05-11 2021-05-25 2021-06-08 2021-06-22 2021-07-06 2021-07-20 2021-08-03 2021-08-17 2021-08-31 2021-09-14 2021-09-28 2021-10-12 2021-10-26 2021-11-09 2021-11-23 2021-12-07 2021-12-21 2022-01-04 2022-01-18

(c) Texas

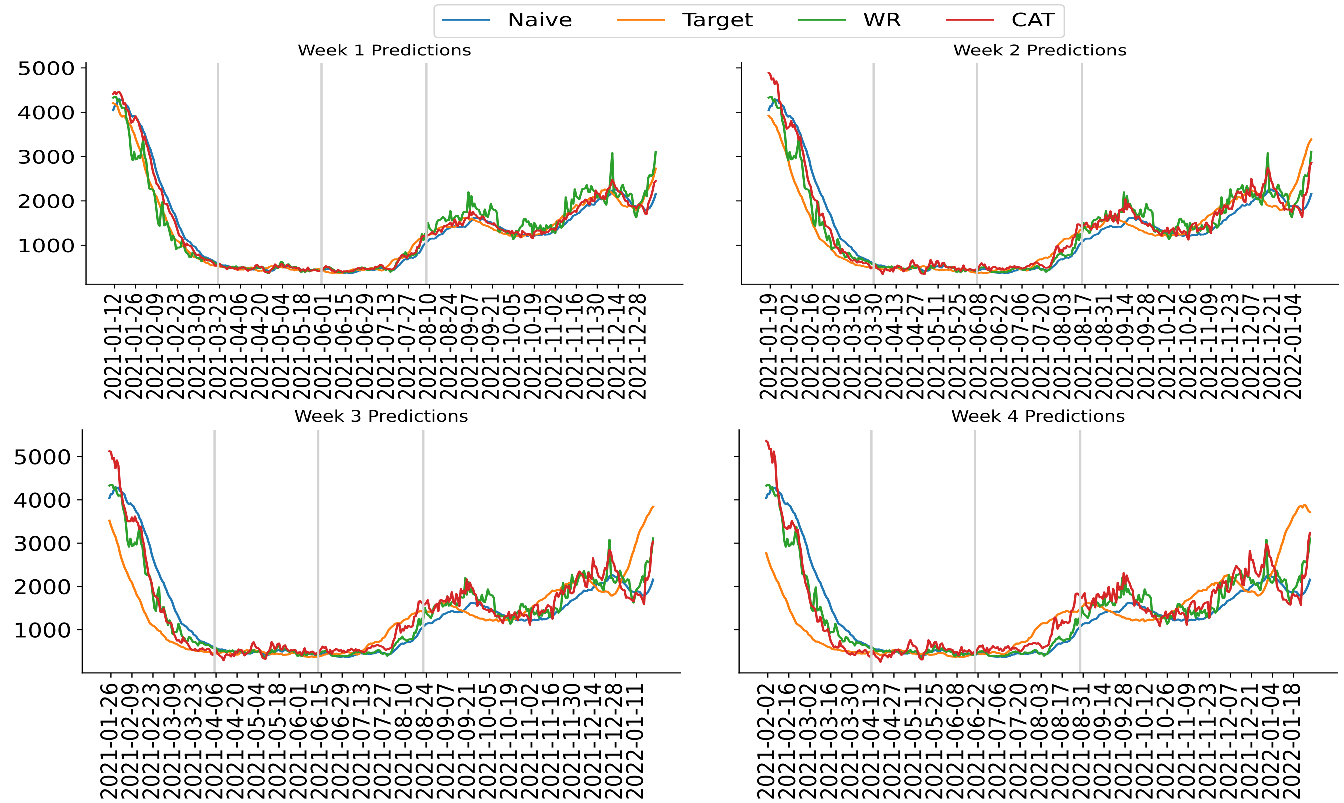

(d) Arizona

Figure S2. Total number of hospitalizations for Week 1 (upper left) predictions, Week 2 (upper right) predictions, Week 3 (lower left) predictions, and Week 4 (lower right) predictions for Illinois, California, Texas, and Arizona. Vertical lines separate different prediction periods as in Table 1 in main article. "Target" is the true reported number of hospitalizations of each state. "CAT" is our proposed model. "WR" is the model without the residual connection. "Naive" is the Naive model prediction.

References

1. Cramer, E. Y. *et al.* The united states covid-19 forecast hub dataset. *medRxiv* DOI: 10.1101/2021.11.04.21265886 (2021).
2. Los Alamos National Laboratory. LANL COVID-19 Cases and Deaths Forecasts. Available at https://covid-19.bsvgateway.org (2020).
3. Luke C Mullany. Pre Smoothed Simulated Linear Pooling for COVID-19 Hospitalization Ensembles (SLPHospEns) model. Available at https://github.com/lmullany/JHUAPL-SLPHospEns (2020).
4. Rodríguez, A. *et al.* Deepcovid: An operational deep learning-driven framework for explainable real-time covid-19 forecasting. *Proc. AAAI Conf. on Artif. Intell.* **35**, 15393–15400 (2021).
5. Johns Hopkins University Applied Physics Laboratory. Bucky model. Available at https://github.com/mattkinsey/bucky (2020).
6. Laboratory for the Modeling of Biological + Socio-technical Systems (MOBS). Analysis of the COVID-19 Pandemic. Available at https://www.mobs-lab.org/2019ncov.html (2020).
7. Lemaitre, J. C. *et al.* A scenario modeling pipeline for COVID-19 emergency planning. *Sci. Reports* **11**, 7534, DOI: 10.1038/s41598-021-86811-0 (2021).
8. Karlen, D. Characterizing the spread of CoViD-19 (2020). arXiv:2007.07156.
9. Srivastava, A., Xu, T. & Prasanna, V. K. Fast and Accurate Forecasting of COVID-19 Deaths Using the SIkJα Model (2020). arXiv:22007.05180.
10. Pei, S. & Shaman, J. Initial Simulation of SARS-CoV2 Spread and Intervention Effects in the Continental US. *medRxiv* DOI: 10.1101/2020.03.21.20040303 (2020). https://www.medrxiv.org/content/early/2020/03/27/2020.03.21.20040303.full.pdf.
11. Ayer, T. *et al.* COVID-19 Simulator. Available at https://covid19sim.org/documents/policy-methods (2021).
12. Johns Hopkins University Applied Physics Laboratory. Gecko model. Available at https://gitlab.jhuapl.edu/panagmj1/gecko-sarima (2020).
